# Process optimisation and genomic analysis of poly(3-hydroxybutyrate) production by *Mycolicibacterium smegmatis* using sugarcane bagasse

**DOI:** 10.1186/s40643-026-01047-y

**Published:** 2026-04-02

**Authors:** Soulayma Hassan, Christian Krohn, Gerardo Aguilar, Alexis Marshall, Andrew S. Ball

**Affiliations:** 1 School of Science, RMIT University, Melbourne, VIC 3083 Australia; 2 ARC Training Centre for the Transformation of Australia’s Biosolids Resource, RMIT University, Bundoora, VIC 3083 Australia

**Keywords:** Biopolymers, Hydrolysate, Lignocellulosic bioconversion, Polyhydroxyalkanoates, Sugar co-utilisation, Whole genome sequencing

## Abstract

This study investigates the optimisation of poly(3-hydroxybutyrate) (PHB) production by *Mycolicibacterium smegmatis* using sugarcane bagasse (SCB) hydrolysate as a low-cost, renewable carbon source. Key fermentation parameters including temperature, pH, agitation, inoculum size and nitrogen supplementation were optimised to enhance biomass growth and PHB accumulation. Under optimised conditions (37 °C, pH 7, 100 rpm, 3% inoculum), *M. smegmatis* achieved a maximum PHB content of 64% of DCW from SCB hydrolysate, corresponding to a PHB titre of ~ 1.0 g L^−1^ and a volumetric productivity of 0.014 g L^−1^ h^−1^ after 72 h of cultivation, with yeast extract identified as the most effective nitrogen source. Thin-layer chromatography (TLC) analysis demonstrated that *M. smegmatis* could not only co-utilise glucose and xylose simultaneously but preferentially consumed xylose, which is a major advantage when processing lignocellulosic biomasses, where xylose is abundant and typically underutilised by many microorganisms. Whole-genome sequencing with Oxford Nanopore Technologies (ONT) confirmed the presence of PHB production genes (*phbA*, *phbB*, *phbC*) and a xylose-utilisation pathway, supporting its metabolic capability. These findings establish *M. smegmatis* as a promising candidate for converting agricultural waste into biodegradable bioplastics, contributing to circular bioeconomy strategies.

**Graphical abstract:**

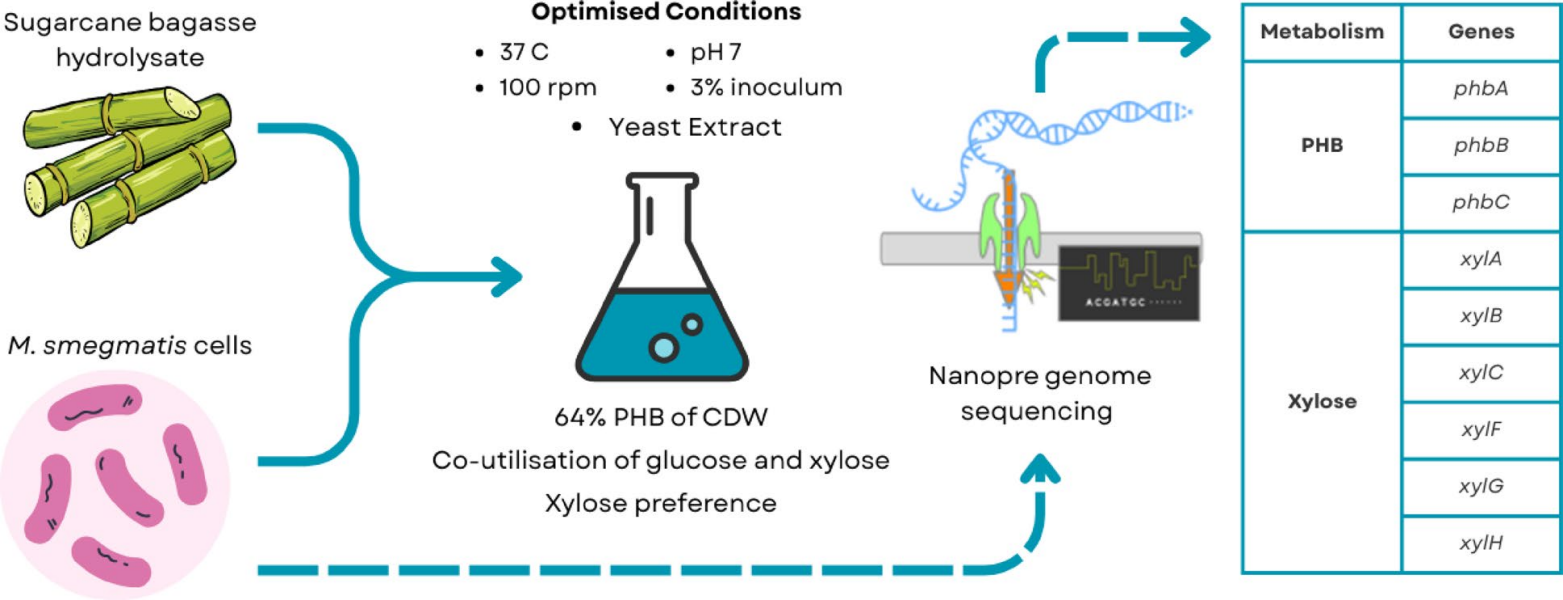

**Supplementary Information:**

The online version contains supplementary material available at 10.1186/s40643-026-01047-y.

## Introduction

In recent years, the development of bioplastics has been extensively explored for its potential contribution in creating a fully sustainable and circular bioeconomy. Due to increasing environmental awareness, the production of bioplastics has been steadily increasing, reaching 1.8 million tonnes in 2023, representing about 0.5% of total plastic output (Mattlar and Ekholm 2025). This trend is particularly evident for bio-based bioplastics which are produced from renewable rather than fossil resources. As a result, bio-based bioplastics exhibit environmental benefits, aligning with some of the United Nations’ (UN) Sustainable Development Goals in terms of a lower carbon footprint and reduced resource depletion compared to fossil-based plastics (Arora and Mishra 2019; Karan et al. 2019).

Polyhydroxyalkanoates (PHAs) are a class of bio-based bioplastics that have attracted great global interest in the last few decades as promising alternatives to conventional plastics due to their biodegradability and production from sustainable raw feedstocks, such as wastewater and lignocellulosic biomass (Muigano et al. 2025). Poly(3-hydroxybutyrate) (PHB) is the most common type of short-chain length (SCL) PHAs, consisting of 3-hydroxy acid monomers containing 3 to 5 carbon atoms. Generally, the synthesis of PHB from fermentable sugars in bacteria occurs via pathway I, where sugars are first converted into acetyl-CoA, then into 3-hydroxybutyryl-CoA, and subsequently polymerised into PHB (Choi et al. 2020). Due to its thermodynamic similarity to polypropylene (PP), PHB has a wide range of applications mainly in eco-friendly packages, biomedical devices and agricultural systems (Briassoulis et al. 2021). Currently, PHB is commercially produced worldwide using pure sugars or edible oils by several companies such as Metabolix (USA), Tianjin Green Bioscience (China), Goodfellow Cambridge (UK) and Bio-on (Italy) (Albuquerque and Malafaia 2018). However, the large-scale production of PHB raises concerns since the feedstocks used compete directly with food resources. Additionally, the high-cost production, mainly the expense of carbon sources, remains a major barrier to PHB commercialisation.

Over recent decades, considerable research has focused on developing cost-effective microbial fermentation processes using inexpensive and renewable carbon sources for sustainable PHB synthesis (Sirohi et al. 2020). Sugarcane bagasse (SCB), an abundant agricultural waste with an annual production of 513 Mt, represents a renewable lignocellulosic feedstock (Hassan et al. 2024). Using SCB for PHB production offers an environmentally friendly, sustainable, and economical approach that avoids competition with food supplies. The direct conversion of mixed sugars (mainly glucose and xylose) derived from SCB into PHB is particularly attractive, as it enables efficient valorisation of both the cellulose and hemicellulose-derived fractions of lignocellulosic biomass, thereby improving overall carbon utilisation and process sustainability. The isolation of new bacteria capable of converting lignocellulosic biomass such as SCB into PHB provides a promising route toward cost-efficient production. *Mycolicibacterium smegmatis* is a rapidly growing mycobacteria, found in soil and environmental samples (Crowley et al. 2024). This non-pathogenic bacterium is widely used as a model organism to investigate the basic biological mechanisms underlying mycobacterial pathogenesis (Sparks et al. 2023). In a previous study conducted by Hassan et al. (2025), M. *smegmatis* was identified and reported for the first time as PHA producing bacteria, with the ability to use not only glucose but also xylose from the SCB hydrolysate. This capability is particularly significant because xylose represents a major fraction of the fermentable sugars released from lignocellulosic biomass, yet it is poorly metabolised by many conventional PHA-producing microorganisms (Zhao et al. 2020). The strain was able to grow and produce PHA directly in non-detoxified SCB hydrolysate, highlighting its tolerance to typical lignocellulosic-derived inhibitors and its suitability for simplified and potentially lower-cost bioprocessing. *M. smegmatis* offers several physiological and bioprocess-relevant advantages that make it an attractive emerging host for PHA production. These include its metabolic versatility and its ability to grow on a wide range of carbon sources (Baloni et al. 2014; Edson, 1951; Titgemeyer et al. 2007). In this previous study the extracted polymer produced by *M. smegmatis* was characterised using FTIR and GC–MS, which unambiguously confirmed the polymer as PHB. Further, *M. smegmatis* exhibited very similar PHB accumulation when cultured on mixed pure sugars (glucose and xylose, 28%) and on SCB hydrolysate (27%), confirming comparable substrate utilisation efficiency, highlighting the potential of using SCB hydrolysate as a low-cost, renewable alternative to refined sugars for PHB production. These findings established the technical feasibility and competitiveness of SCB hydrolysate as a carbon source and provided the basis for the present work. Although *M. smegmatis* has exhibited the capability to synthesise PHB from SCB hydrolysate, the observed accumulation level (27%) was comparatively low relative to high-PHB-accumulating bacterial species. Therefore, the present study aims to optimise bioprocess parameters and assess the effects of various nitrogen sources to primary enhance intracellular PHB accumulation (PHB % of DCW), while also evaluating biomass formation and PHB titre (g L^−1^) as secondary performance indicators to reflect overall production efficiency. In addition, the utilisation patterns of glucose and xylose in mixed substrates by *M. smegmatis* was examined using Thin Layer Chromatography (TLC). Further, whole-genome sequencing (WGS) analysis was conducted to elucidate the metabolic pathways involved in PHB biosynthesis in *M. smegmatis*, including those associated with xylose metabolism.

## Materials and methods

### Sugarcane bagasse preparation and pretreatment

SCB was supplied from Tully sugar mill in Queensland, and pretreated as described by Hassan et al. (2025). Briefly, the dried bagasse was chopped into small pieces for less than 2 cm in size, ground and sieved though a 0.35 mm mesh. The pretreatment process involved soaking SCB in a 2% (w/v) sodium hydroxide (NaOH) solution with a solid-to-liquid ratio of 1:10 for 30 min at 121 °C. Pretreated SCB was filtered and rinsed with milli-Q water until reaching a neutral pH and dried at 65 ℃ for 24 h before enzymatic hydrolysis. To convert the lignocellulosic material into fermentable sugars, enzymes from *Aspergillus niger* cellulases (≥ 0.3 units/mg solid) and xylanase from recombinant *Aspergillus oryzae* (≥ 2500 units/g), both obtained from Sigma Aldrich, USA, in a 2:1 ratio were added. The enzyme mixture, with filter paper unit (FPU) activity of 25 IU per gram of SCB, was added to 25 mL of sodium acetate buffer (50 mM, pH 5) containing the SCB. The mixture was incubated at 50 °C and agitated at 150 rpm on a rotary shaker for 40 h. Post-hydrolysis, enzyme activity was halted by heating the mixture to 100 °C for 10 min. The hydrolysed solution was then cooled and filtered under vacuum using Whatman No.1 filter paper. The concentration of sugars in the resulting supernatant was measured using the dinitrosalicylic acid (DNS) method, with glucose as the reference standard (Miller 1959).

The hydrolysate was stored at 4 °C for and used to assess PHB production under optimised conditions.

### Bacteria and culture media

*M. smegmatis* was isolated previously from rotten SCB as described by Hassan et al. (2025). The initial growth medium used to grow the inoculum was a nutrient broth medium containing peptone (15 g L^− 1^), sodium chloride (6 g L^− 1^), yeast extract (3 g L^− 1^) and glucose (1 g L^− 1^). The inoculum was cultured in an Erlenmeyer flask for 48 h at 37 ℃ and 100 rpm. The optical density of the inoculum at 600 nm (OD₆₀₀) was measured using a spectrophotometer (UV-1800, Shimadzu, Kyoto, Japan) with a 1 cm path-length cuvette. The inoculum used for all experiments was standardised to an OD₆₀₀ of 1.0. For inoculum preparation, cells were harvested and cell pellets were collected by centrifugation at 10,000× g for 10 min at 4 °C, followed by two washing steps with Milli-Q water prior to resuspension and inoculation. The culture medium used for the optimisation of PHA production was a minimal salt medium supplied with a mixture of glucose and xylose (2:1) and consisted of NaH_2_PO_4_ (3.6 g L^− 1^), Na_2_HPO_4_ (2.84 g L^− 1^), K_2_SO_4_ (3.49 g L^− 1^), NaOH (0.4 g L^− 1^), yeast extract (0.2 g L^− 1^), MgSO_4_·7H_2_O (0.39 g L^− 1^), CaCl_2_ (0.062 g L^− 1^), (NH_4_)_2_SO_4_ (0.1 g L^− 1^), CuSO_4_·5H_2_O (0.005 g L^− 1^), ZnSO_4_·7H_2_O (0.024 g L^− 1^), MnSO_4_·H_2_O (0.024 g L^− 1^), FeSO_4_·7H_2_O (0.15 g L^− 1^), and sugar mixture (20 g L^− 1^). (NH_4_)_2_SO_4_ was used only as the standard nitrogen source during the preliminary optimisation stage. After the optimal cultivation conditions were established, (NH_4_)_2_SO_4_ was no longer used by default. Instead, only the selected nitrogen source under investigation (peptone, yeast extract, NH_4_Cl or (NH_4_)_2_SO_4_) was added to the medium during the nitrogen-source optimisation experiments.

### Optimisation of experimental conditions and nitrogen supplement selection

Production of PHA was optimised using a range of incubation periods (24, 48, 72 and 96 h), temperatures (25, 30, 37 and 40 ℃), agitation rates (static, 50, 100 and 150 rpm), pH values (6,7, 8 and 9) and inoculum concentrations (1,2,3 and 5%) in 250 mL Erlenmeyer flasks. The final working volume in each flask was 50 mL, corresponding to a fill volume of 20% and a flask-to-working-volume ratio of 5:1 (250 mL flask/50 mL culture). Except for the parameter to be optimised, other experimental conditions were kept the same as followed: for 48 h, 37 ℃, 100 rpm with pH 7 and 1% inoculum. Bacterial growth and PHA accumulation were studied as described in 2.4. All experiments were performed in triplicate.

Under optimised conditions, SCB hydrolysate was utilised for PHB production. To enhance bacterial growth and PHA accumulation, various nitrogen supplements including peptone, ammonium sulfate ((NH_4_)_2_SO_4_), ammonium chloride (NH_4_Cl) and yeast extract were added to the hydrolysate (1% (*w/v*)) and assessed for the highest production of PHA.

### PHA production and bacterial growth analysis

To determine dry cell weight (DCW), cell pellets were harvested into pre-weighed tubes by centrifugation for 10 min at 10,000 x g, 4 °C, washed 2 times with milli-Q water and freeze-dried for 12 h using a lyophiliser (ALPHA 1–2 LDplus, Osterode, Germany). DCW was calculated by subtracting the weight of the empty tube from the weight of the same tube containing the lyophilised pellet, with the result expressed in grams per litre (g L^−1^). PHA was extracted from the lyophilised cells using a combination of sodium hypochlorite and chloroform, following the method described by Hahn et al. (1994). Briefly, 2 mL each of 30% (v/v) sodium hypochlorite and chloroform (in a 1:1 ratio) were added to the dry pellet. This mixture was incubated at 30 °C for 5 h on a rotary shaker at 150 rpm. After incubation, three separate layers formed. The bottom layer, which contained the chloroform and dissolved PHA, was carefully collected into a pre-weighed tube. To precipitate the PHA, chilled methanol with four times the volume of the collected chloroform- PHA solution was added, and the mixture was incubated at − 20 °C for 30 min. The PHA appeared as a white solid precipitate, which was then dried in a fume hood and weighed. The percentage of PHA accumulation was calculated using the following formula:

$$ \begin{aligned} {\text{PHA accumulation }}\left( \% \right) = & {\text{Dry weight of PHA }}\left( {g{\text{ }}L^{{ - {\text{ }}1}} } \right)/ \\ & {\mathrm{DCW}}\left( {g{\text{ }}L^{{ - {\text{ }}1}} } \right){\text{ }} \times {\text{ }}100 \\ \end{aligned} $$

### Sugar utilisation investigation using thin layer chromatography (TLC)

To investigate the sugar preference of *M. smegmatis*, the use of glucose and xylose was monitored using TLC. The total sugar concentration of the mixture was 20 g L⁻¹, consisting of 10 g L⁻¹ glucose and 10 g L⁻¹ xylose (1:1, w/w). The mixture was inoculated with 1% *M. smegmatis*, and culture supernatants were collected at 48, 72, 96 and 120 h intervals during growth for residual glucose and xylose.

The procedure was performed as described by Farag (1979). Aliquots of 2 µL of culture supernatant and standard solutions were spotted onto silica gel plates and air-dried for 30 min. Plates were developed in the ascending direction using a solvent system consisting of a mixture of chloroform, acetic acid and water with a ratio of 3:3.5:0.5 by volume, respectively. Once the solvent front had travelled 12.5 cm (after approximately 90 min), the plates were removed and air-dried for 30 min. To improve resolution, the plates were redeveloped in the same solvent to the same distance, then air-dried for further 30 min. A prepared solution of 1 g diphenylamine and 1 mL of aniline in 100 mL of acetone, mixed with 85% orthophosphoric acid (10:1 v/v) was sprayed evenly on the dried plates. Spots were visualised by gentle heating. The utilisation of glucose and xylose was assessed by calculating the relative intensity of each spot to initial intensity of glucose and xylose spots using Phoretix 1D software (version 628, TotalLab Ltd.).

### Genomic investigation for PHA production and xylose utilisation

Genomic DNA was extracted using a DNA Extraction & Purification Kit (New England Biolabs, Ipswich, MA, USA) following the manufacturer’s protocol (New England Biolabs). DNA quantity and purity were initially assessed using the NanoDrop Lite Spectrophotometer (Thermo Fisher Scientific, Waltham, MA, USA), and precise DNA concentrations were determined using the Qubit™ Fluorometric Quantification System (Invitrogen, Waltham, MA, USA).

Whole-genome sequencing (WGS) was performed on a MinION Mk1D (Oxford Nanopore Technologies, Oxford, UK). Duplicate genome libraries were prepared using the Native Barcoding Kit 24 V14 (SQK-NBD114.24) according to the manufacturer’s protocol (Oxford Nanopore Technologies, 2025). The pooled library was subsequently loaded onto a R10.4.1 flow cell (FLO-MIN114), sequenced and basecalled in real time using Dorado v7.9.8 in super-accurate mode until sufficient data was acquired (minimum Q ≥ 15). This yielded a total of 5.58 Gb at a median read quality of Q24 across 2,722,500 reads (N50 = 4.45 kb) with a fraction of reads at lengths from 10 to 616 kb.

Two long-read assemblies were produced, one de novo and one reference-guided (*Mycolicibacterium smegmatis* accession CP000480.1) using the EPI2ME wf-bacterial-genomes workflow (EPI2ME Labs, Oxford Nanopore Technologies; https://github.com/epi2me-labs/wf-bacterial-genomes). The workflow concatenated and quality-filtered reads, assembled genomes with Flye (v2.9.5-b1801), polished them with Medaka (v2.0.0), and generated automated annotations via Prokka (v1.14.5). The assemblies produced high-contiguity bacterial genomes with > 100× coverage, dominated by a single circular 7.34 Mb contig at uniform read-mapping profiles, supporting a confident reference match and providing genomes well suited for downstream taxonomic and functional analyses. The genome assembly has been deposited at GenBank under the accession JBSZQJ000000000.

Post-assembly quality assessment and refinement were further conducted using tools within the Galaxy Australia platform (https://usegalaxy.org.au/). QUAST (Galaxy Version 5.3.0 + galaxy1) was employed to evaluate assembly metrics, including genome completeness and contamination with CheckM2 (Galaxy Version1.1.0 + galaxy0). FastANI (Galaxy Version 1.3) was subsequently used for taxonomic identity confirmation and average nucleotide identity (ANI) comparison against the closest reference genome (*Mycolicibacterium smegmatis* mc²155 accession CP000480.1).

Genome annotation and metabolic pathway reconstruction were further performed using the Rapid Annotation using Subsystem Technology (RAST) pipeline using Seed Viewer (https://rast.nmpdr.org/) with the focus on genes associated with polyhydroxyalkanoate (PHA) biosynthesis pathways and xylose utilisation by *M. smegmatis*.

## Results and discussion

### Optimisation of PHB production using *M. smegmatis*

#### Effect of incubation temperature and pH on PHB production

**Fig. 1:**
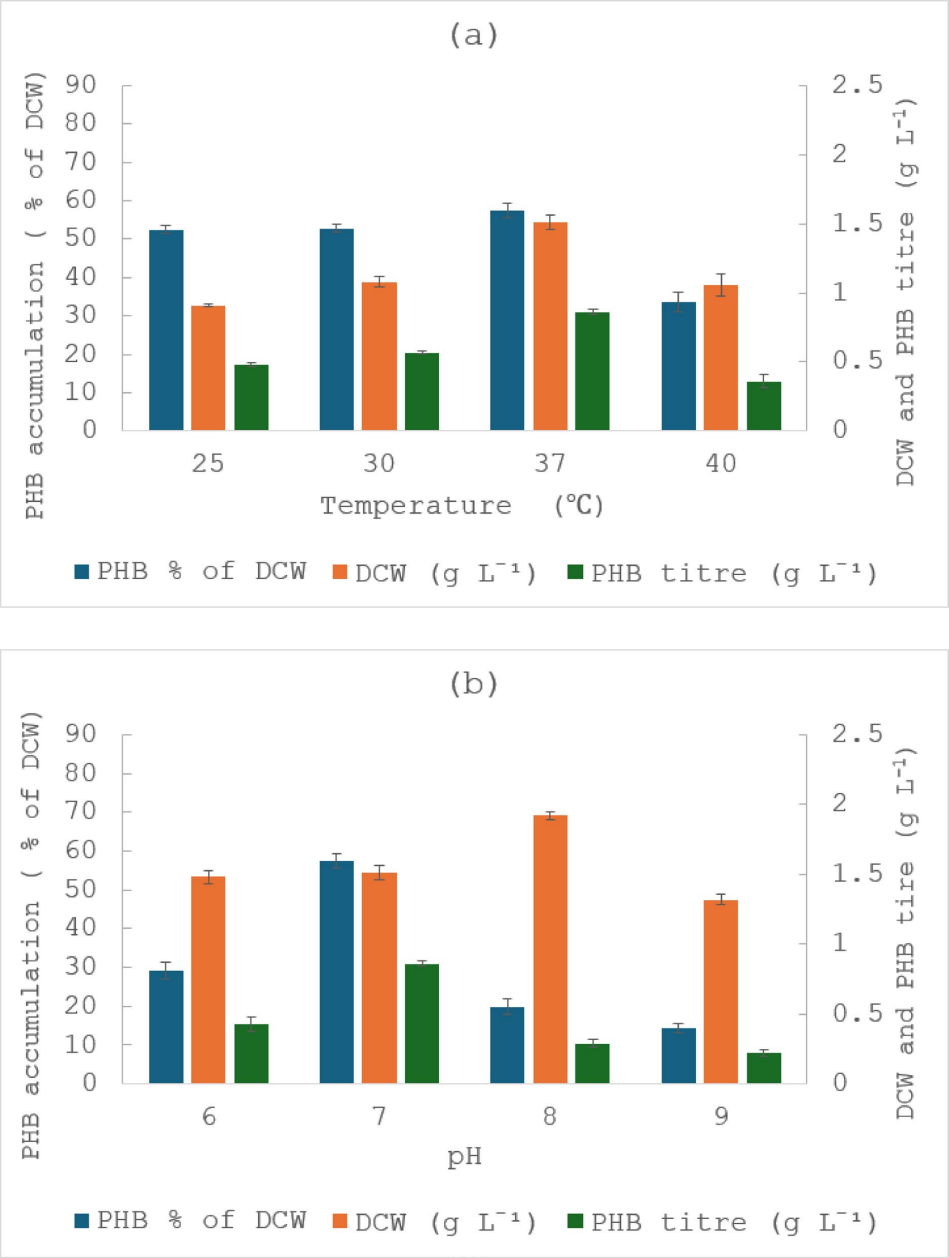

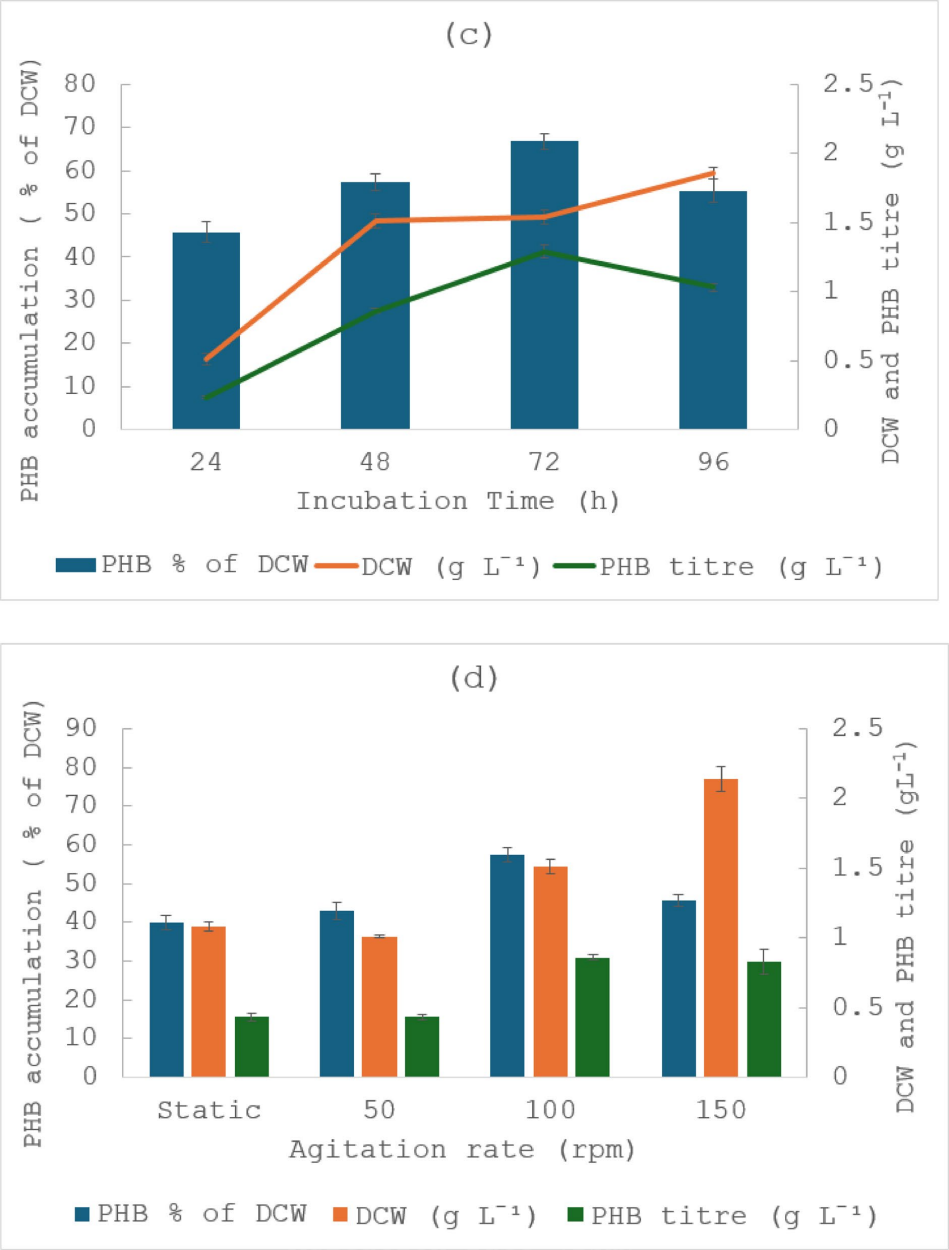

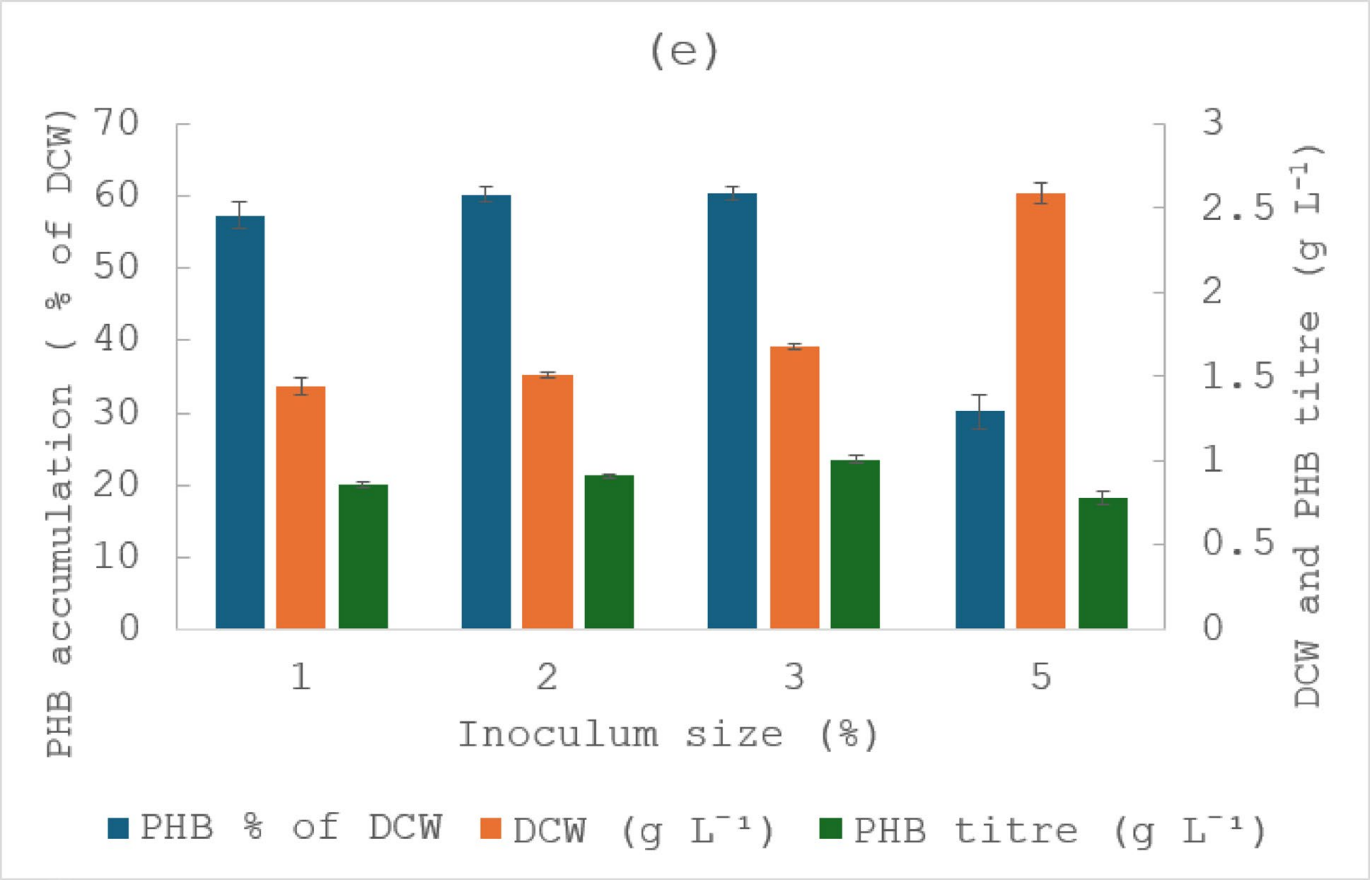
Effect of **a** incubation temperature, **b** pH, **c** incubation time, **d** agitation rate and **e** inoculum size on dry cell weight (g L^− 1^) and PHB accumulation (% of DCW) and PHB titre (g L^− 1^) by *M. smegmatis*. The results are the means of three replicates, with error bars shown as ± standard deviation

The physiochemical conditions surrounding bacteria play a critical role in their growth and metabolism (Wang et al. 2021). This study aimed to optimise the fermentation conditions to enhance PHB production. Figure 1a and 1.b shows the DCW (g L^− 1^) and PHB accumulation (% of DCW) and PHB titre (g L^− 1^) as function of different temperatures and pH. Incubation temperature and pH are key factors affecting the production of PHA in bacteria (Mahato et al. 2021; Saratale et al. 2021; Sriyapai et al. 2022). In response to various incubation temperatures, *M. smegmatis* showed an increase in DCW and PHB accumulation from 25 °C to 37 °C. Maximum DCW and PHB accumulation were reached at 37 °C with values of 1.51 g L^− 1^ and 57.4%, respectively. However, bacterial growth and PHB accumulation were found to decrease with further increase in temperature. This may have been due to reduced enzymatic activity, including the ones responsible for PHB production at these temperatures. *M. smegmatis* is known for its adaptability to a broad pH range, typically growing between pH 5.0 and 7.4 (Apiyo et al. 2022). In the present study, *M. smegmatis* demonstrated active growth at pH 6, 7, and 8, indicating a wider pH tolerance than previously documented. Although the highest DCW was observed at pH 8, highest PHB accumulation occurred at pH 7. This suggests that conditions favouring cell growth do not necessarily maximise carbon flux towards PHB accumulation. A neutral pH is more favourable for balanced cellular metabolism and for enzyme activity in *M. smegmatis*, including PHB synthesis, whereas slightly alkaline conditions may promote biomass formation but redirect metabolic flux towards growth and maintenance rather than polymer accumulation. Similar findings have been reported in other PHB producing bacteria, such as *Cupriavidus necator* and *Bacillus megaterium*, where maximum PHA production were seen with pH 7 (Mittal et al. 2024; Mohanrasu et al. 2020).

#### Effect of incubation time on PHB production

The influence of incubation time on DCW and PHB accumulation was evaluated over 96 h (Fig. 1c). The results show a steady increase in bacterial mass from 0.51 g L^− 1^ at 24 h to 1.86 g L^− 1^ at 96 h, with the fastest growth occurring between 24 and 48 h, indicating active cell proliferation during this period. PHB accumulation increased from 45.8% at 24 h to reach a maximum of 66.9% at 72 h, followed by a decrease to 55.4% at 96 h. These observations suggest that PHB accumulation in *M. smegmatis* occurred predominantly during the mid-exponential to early stationary phase, with the highest yield achieved at 72 h of incubation. The subsequent decline in PHA percentage after 72 h may be attributed to polymer degradation or consumption by cells as energy source when needed. Different optimal incubation times for PHA accumulation have been reported in the literature, such as 48 h for *Lysinibacillus* sp., 72 h for *Pseudomonas aeruginosa* and 120 h for *Erythrobacter aquimaris* (Mahato et al. 2021; Mostafa et al. 2020; Saratale et al. 2021).

#### Effect of agitation rate on PHB production

The effect of agitation rate on DCW and PHB accumulation was examined under static and agitated conditions (Fig. 1d). DCW increased progressively with agitation, from approximately 1.08 g L^− 1^ under static conditions to 2.14 g L^−1^at 150 rpm, reflecting the positive effect of agitation on oxygen solubilisation and nutrient mixing on bacterial growth. PHB accumulation followed a different trend as it remained relatively constant under static and 50 rpm conditions, then rose to a maximum of 57% before it declined at 150 rpm. These findings indicate that a moderate agitation of 100 rpm provides optimal balance between oxygen transfer and metabolic stress, supporting both cell growth and PHB accumulation in *M. smegmatis*.

#### Effect of inoculum size on PHB production

Inoculum size is another important parameter in the optimisation process. *M. smegmatis* was inoculated into the production medium with varying inoculum size of 1%, 2%, 3% and 5% (v/v) (Fig. 1e). The results showed that increasing inoculum size influenced both cell growth and PHB production. DCW increased from 1.44 g L^− 1^ at 1% inoculum to a maximum of 2.59 g L^− 1^ at 5%. However, PHB accumulation increased slightly from 57.4% at 1% to 60.4% at 3%, followed by a sharp decline to 30.2% at 5%. Increasing inoculum size resulted in higher DCW but lower PHB accumulation at 5% compared with 3%. High initial cell density is known to accelerate nutrient, which can shorten the effective production phase and reduce the availability of carbon excess required to trigger intracellular PHB synthesis (Ali et al. 2024). An intermediate inoculum size (3%) provided a more favourable balance between biomass formation and the metabolic conditions required for PHB accumulation.

### PHB production by *M. smegmatis* using sugarcane bagasse hydrolysate under optimised conditions

**Fig. 2:**
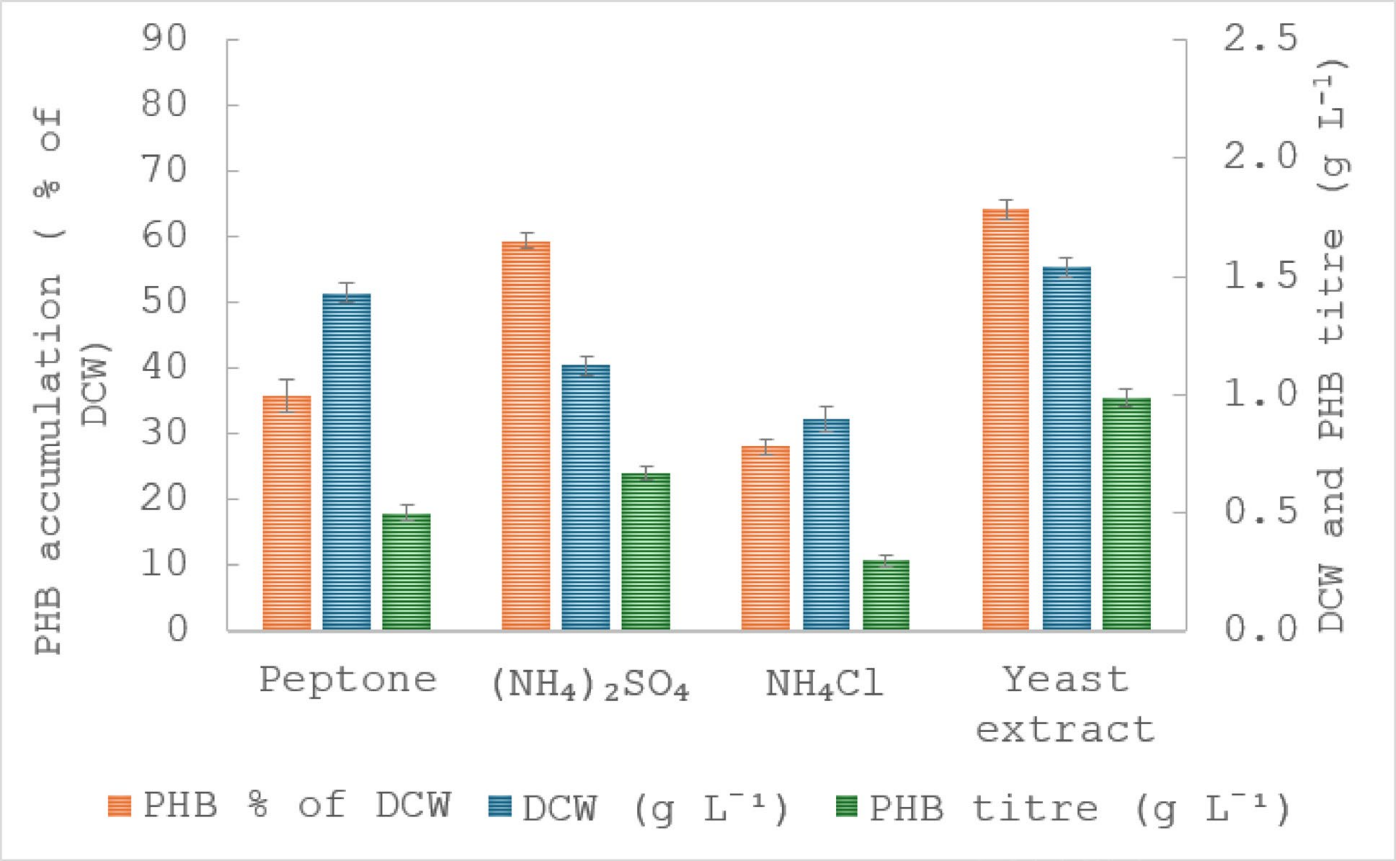
Effect of different nitrogen sources on *M*. *smegmatis* growth (g L^− 1^), PHB accumulation (% of DCW) and PHB titre (g L^− 1^) using SCB hydrolysate. The results are the means of three replicates, with error bars shown as ± standard deviation

A suitable nitrogen source plays a major role in bacterial growth and metabolism (Alsafadi et al. 2020). PHA biosynthesis in bacteria is sensitive to nitrogen, and the majority of PHA-producing bacteria have different preferences of nitrogen sources, such as nitrate, ammonia, urea etc. (Benesova et al. 2017). Therefore, the selection of nitrogen sources is important to enhance PHA accumulation. In this study, the effect of different organic (peptone and yeast extract) and inorganic nitrogen sources ((NH_4_)_2_SO_4_ and NH_4_Cl) on cell growth and PHB accumulation by *M. smegmatis* utilising SCB hydrolysate was examined under optimised culture conditions (Fig. 2). Among the tested sources, yeast extract supported the highest PHB accumulation with an accumulation of 64.2%, followed by ammonium sulfate ((NH_4_)_2_SO_4_) at 59.4%, peptone at 36%, and ammonium chloride (NH_4_Cl) at 30%. In contrast, DCW was greatest when peptone was used (1.4 g L^− 1^), followed by yeast extract (1.24 g L^− 1^), (NH_4_)_2_SO_4_ (1.1 g L^− 1^), and NH_4_Cl (0.9 g L^− 1^). These findings suggest that organic nitrogen sources, particularly peptone and yeast extract, supported higher biomass formation, likely due to the presence of readily assimilable amino acids and additional growth factors. In contrast, inorganic nitrogen sources ((NH_4_)_2_SO_4_ and NH_4_Cl) resulted in lower cell dry weight but, in the case of (NH_4_)_2_SO_4_, enabled relatively high PHB accumulation. Therefore, yeast extract was identified as the most suitable nitrogen source for maximising PHB production by *M. smegmatis* using SCB hydrolysate as the primary carbon substrate. All nitrogen sources were added at a concentration of 1% (w/v). However, the actual amount of N added varied according to the N composition of the substrate (Table S1). In particular, the inorganic salts supplied approximately 2–3 times more nitrogen per litre than the complex organic sources. In addition, peptone and yeast extract also contributed non-nitrogen nutrients (e.g. carbon, vitamins and growth factors), which may have influenced bacterial growth and PHB accumulation independently of nitrogen availability.

The utilisation of sugars derived from sugarcane bagasse is highly significant for improving both the economic viability and sustainability of PHB production, as it enables the replacement of refined sugars with an abundant, low-cost agricultural residue (Zytner et al. 2023). In batch fermentation using SCB hydrolysate as carbon source and yeast extract as nitrogen source with 3% inoculum size, *M. smegmatis* exhibited maximum PHB accumulation of 64% under agitation of 100 rpm, at 37 °C, pH 7 after 72 h of fermentation, corresponding to a PHB titre of approximately 1.0 g L^−1^ and a volumetric productivity of 0.014 g L^−1^ h^−1^. Although the DCW of *M. smegmatis* obtained in this study was relatively low compared to other reports, the percentage of PHB accumulation within the cells was high (Table 1). This suggests that the metabolic flux of *M. smegmatis* was mainly directed towards PHB synthesis rather than cell proliferation. Furthermore, potential inhibitory compounds derived from SCB pretreatment, such as furfural, hydroxymethylfurfural (HMF) and acetic acid, which are commonly reported in alkaline pretreated lignocellulosic hydrolysates, could have contributed to slower cell proliferation (Kovalcik et al. 2018; Ladeira-Ázar et al. 2019; Lyu et al. 2025; Vieira et al. 2020). To address this issue in future studies, the optimisation process could focus on implementing a two-stage cultivation strategy where initially cell proliferation is promoted under nutrient-sufficient conditions, followed by PHB biosynthesis induction under controlled nutrient limitation. In addition, future work may explore co-culture-based strategies, in which *M. smegmatis* is cultured alongside complementary microorganisms capable of enhancing substrate conversion efficiency or modulating the extracellular environment, with the aim of improving overall biomass formation and PHB productivity from lignocellulosic-derived sugars. *M. smegmatis* has only recently been identified as a potential PHA-producing bacterium; consequently, limited information is currently available regarding its metabolic pathways and physiological responses during PHB accumulation. Further studies are therefore needed to elucidate its biosynthetic mechanisms and develop strategies to maximise both biomass and PHB production under controlled conditions.

**Table 1:** Comparison of *M. smegmatis* growth (g L^− 1^) and PHA accumulation (% of DCW) and PHA titre (g L^− 1^) with the literature using SCB hydrolysate in batch system

Microorganism	Dry cell weight (g L^− 1^)	PHA accumulation (% of DCW)	PHA titre (g L^− 1^)	Reference
*Bacillus megaterium* PNCM 1890	4.9	41	2	(Barrameda et al. 2023)
*Bacillus* sp	9	56	5	(Getachew and Woldesenbet 2016)
*Burkholderia glumae MA13*	0.61	15	9	(de Paula et al. 2020)
*Klebsiella* *pneumoniae* G1	22.5	40	9	(Siripurapu et al. 2022)
*Lysinibacillus* sp.RGS	8.7	62	5	(Saratale et al. 2021)
*Mycolicibacterium smegmatis*	0.6	27	0.2	(Hassan et al. 2025)
*Mycolicibacterium smegmatis*	1.5	64	1	This study

### Comparison of glucose and xylose utilisation by *M. smegmatis* using thin layer chromatography (TLC)

**Fig. 3:**
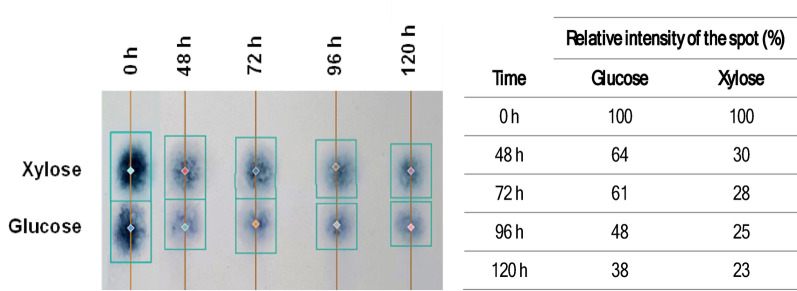
Image analysis of glucose and xylose spots on TLC at different time intervals. The intensity of the spots is reported in the table on the right

In the previous study, *M. smegmatis* was reported for its ability to produce PHB using glucose and xylose as carbon source (Hassan et al. 2025). In this study, TLC was employed to investigate the ability of *M. smegmatis* to utilise both glucose and xylose simultaneously and to determine its sugar utilisation preference. The relative intensity of each sugar spot, which represents the amount of residual sugar, was measured at different incubation times (48–120 h) and expressed as a percentage of the initial concentration (Fig. 3). This approach was used as a qualitative and semi-quantitative tool to support interpretation of substrate utilisation behaviour from a mixture of pure sugars, rather than for detailed quantitative carbon balance analysis. The results showed a gradual decrease in the relative intensity of both glucose and xylose with increasing incubation time, indicating that *M. smegmatis* could metabolise both sugars derived from SCB hydrolysate. At 48 h, the relative intensity of glucose and xylose was 64% and 30%, respectively. By 120 h, only 38% of glucose and 23% of xylose were detected. This pattern suggests that, starting from the same concentration of glucose and xylose, *M. smegmatis* preferentially consumed xylose over glucose when both sugars were available in the medium. Such preferential utilisation of xylose by *M. smegmatis* contrasts with the typical sugar utilisation pattern observed in many bacteria (one exception is *Schlegelella thermodepolymerans*), which generally favour glucose due to catabolite repression (Kourilova et al. 2020; Vasylyshyn et al. 2020; Zhao et al. 2020). This might be considered advantageous for the utilisation of lignocellulosic hydrolysates, where xylose represents a major component (Ashby 2020). Even *Burkholderia sacchari* DSM 17,165, the most efficient PHA-producing microorganism from xylose-rich substrates, prefers glucose over xylose (Raposo et al. 2017). The ability of *M. smegmatis* to co-utilise and even favour xylose over glucose highlights its potential as a robust candidate for bioconversion processes and PHB production from mixed sugar feedstocks.

### Genomic analysis of *M. smegmatis* for the synthesis of PHB and xylose utilisation

Given that *M. smegmatis* is newly reported for PHB production, a genomic overview was performed to identify key genes supporting its biopolymer production potential. Xylose utilisation pathway was also explored, allowing assessment of its potential to integrate pentose sugars into central metabolism for biopolymer synthesis. Several tools were used to assess assembly quality, contiguity and completeness prior to downstream genomic interpretation. QUAST analysis showed that the de-novo assembly consisted of 19 contigs, with 18 contigs > 1 kb and 7 contigs > 10 kb. Notably, a single dominant contig measured 7.34 Mb, resulting in an N50 of 7,344,559 bp and L50 of 1. These values indicate that the assembly is highly contiguous, with most genomic content captured in one large, well-resolved contig. The total genome size was 7.97 Mb, which is slightly larger than the typical *M. smegmatis* genome size of 6.9–7.0 Mb (Dovbnya et al. 2022). This modest increase in size is likely due to strain-specific genomic expansions, the presence of mobile genetic elements, or partially uncollapsed repetitive regions commonly associated with long-read assemblies (Vale et al. 2022). The genome exhibited a GC content of 67.1%, matching the expected high-GC profile of *Mycolicibacterium* species (61–71%) (Morgado and Vicente, 2021). CheckM2 analysis demonstrated 100% completeness and only 0.73% contamination, indicating that the assembly successfully captured the full genomic complement with minimal artefacts. FastANI confirmed the taxonomic identity of the isolate, showing 98.04% average nucleotide identity (ANI) to the reference genome *M. smegmatis* MC²155 (CP000480.1), exceeding the 95% species threshold and validating the isolate as a close relative of the model strain.

A total of 7963 coding genes were annotated by RAST. Of them, a total of 1481 (19%) protein-coding genes were functionally allocated into 28 subsystems. Many microorganisms produce PHA from acetyl-CoA through the sequential activity of three core enzymes. First, *β*-ketoacyl-CoA thiolase (*PhaA*) catalyses the condensation of two acetyl-CoA and acetyl-propionyl-CoA. The resulting acetoacetyl-CoA is then reduced to hydroxyacyl-CoA by acetoacetyl-CoA reductase (*PhaB*). Finally, PHA synthase (*PhaC*) polymerises hydroxyacyl-CoA into poly(3-hydroxyalkanoate), releasing CoA in the process (Tyagi et al. 2022). In this study, genomic analysis of *M. smegmatis* revealed the presence of key genes for PHB production: 3-ketoacyl-CoA thiolase (*phbA*), acetoacetyl-CoA reductase (*phbB*) and PHA synthase (*phbC*) as shown in Table 2. Other enzymes were also identified such as enoyl-CoA hydratase (*PhaJ)*.

The genome of *M. smegmatis* also contained genes encoding for key xylose metabolism enzymes via the xylose isomerase pathway. This pathway includes the conversion of xylose into D-xylulose via the action of xylose isomerase and, consequently, the phosphorylation of D-xylulose into D-xylulose-5-phosphate by xylulokinase, which is further metabolised in the pentose phosphate pathway (PPP), also detected in *M. smegmatis* (Nieves et al. 2015). Six genes responsible for xylose utilisation were identified, including xylose isomerase (*xylA*), xylulose kinase (*xylB*), and three genes responsible for sugar transport including D-xylose ABC transporter substrate-binding protein (*xylF*), D-xylose ABC transporter ATP-binding protein (*xylG*), D-xylose ABC transporter permease protein (*xylH*). The detection of key genes associated with PHB production, together with the presence of a comprehensive xylose-utilisation system, suggests that *M. smegmatis* possesses the metabolic capacity to channel pentose sugars into central carbon metabolism, which can subsequently supply precursors (such as acetyl-CoA) for the PHB biosynthetic pathway. This is further supported by previous findings showing that *M. smegmatis* can use xylose as a sole carbon source for PHB production (Hassan et al. 2025).

**Table 2:** The important enzymes involved in PHB production and xylose utilisation by *M. smegmatis* in this study

	EC Number	Enzyme
Polyhydroxybutyrate metabolism	EC 2.3.1.9	Acetyl-CoA acetyltransferase
	–	Polyhydroxyalkanoic acid synthase
	EC 2.3.1.16	3-ketoacyl-CoA thiolase
	EC 6.2.1.16	Acetoacetyl-CoA synthetase
	EC 1.1.1.157	3-hydroxybutyryl-CoA dehydrogenase
	EC 1.1.1.35	3-hydroxyacyl-CoA dehydrogenase
	EC 4.2.1.55	3-hydroxybutyryl-CoA dehydratase
	EC 1.1.1.36	Acetoacetyl-CoA reductase
	EC 4.2.1.17	Enoyl-CoA hydratase
	EC 5.1.2.3	3-hydroxybutyryl-CoA epimerase
Xylose metabolism	EC 5.3.1.5	Xylose isomerase
	EC 2.7.1.17	Xylulose kinase
	–	Xylose-responsive transcription regulator, ROK family
	–	D-xylose ABC transporter, substrate-binding protein XylF
	7.5.2.10	D-xylose ABC transporter, ATP-binding protein XylG
	–	D-xylose ABC transporter, permease protein XylH

## Conclusion

This study highlights *M. smegmatis* as a promising candidate for PHB production from sugarcane bagasse hydrolysate. Under optimised conditions, the strain achieved high PHB accumulation of 64% after 72 h, with yeast extract emerging as the most effective nitrogen source for enhancing production. A key advantage observed was the bacterium’s ability to co-utilise both glucose and xylose with xylose as preference, enabling efficient conversion of mixed-sugar lignocellulosic substrates and improving overall carbon utilisation. Whole-genome sequencing further confirmed the presence of key PHB biosynthetic genes and a xylose-isomerase pathway, supporting its metabolic capability to process pentose-rich hydrolysates. Future research should explore strategies to increase biomass yield and PHB productivity from lignocellulosic-derived sugars such as two-stage cultivation and co-culture strategies or apply metabolic engineering to boost PHB flux. Additionally, bioreactor optimisation and scale-up studies will be essential for evaluating commercial viability and advancing *M. smegmatis* as a sustainable bioplastic production platform within a circular bioeconomy.

## Supplementary Information

Below is the link to the electronic supplementary material.

Supplementary Material 1

## Data Availability

The datasets used during the current study are available from the corresponding author on reasonable request.
