# Supplementary material for: Process optimisation and genomic analysis of poly(3-hydroxybutyrate) production by Mycolicibacterium smegmatis using sugarcane bagasse
Source: Bioresour Bioprocess. 2026 Apr 2;13(1):48. doi: 10.1186/s40643-026-01047-y (PMC13046884; doi:10.1186/s40643-026-01047-y)
Supplement: Supplementary file 1 — Supplementary Material 1 [file 40643_2026_1047_MOESM1_ESM.docx]

**Process optimisation and genomic analysis of poly(3-hydroxybutyrate) production by** ***Mycolicibacterium smegmatis* using sugarcane bagasse**

By Soulayma Hassan, Christian Krohn, Gerardo Aguilar, Jr., Alexis Marshall and Andrew S. Ball

**Supplementary Material**

**Table S1.** Estimated nitrogen supplied by each nitrogen source at 1% (w/v) with a working volume of 50 mL

| Nitrogen source | Supplier* specification | Estimated nitrogen supplied (g N L⁻¹) | Estimated nitrogen supplied per flask (50 mL) |
| --- | --- | --- | --- |
| Peptone | Amino-nitrogen ≥ 4.5%; Total nitrogen ≥ 9.5% | ≥ 0.95 g N L⁻¹ *(amino-N ≥ 0.45 g N L⁻¹)* | ≥ 0.0475 g N (47.5 mg N) |
| Yeast extract | Amino-nitrogen ≥ 4.5%; Total nitrogen ≥ 10% | ≥ 1.00 g N L⁻¹ *(amino-N ≥ 0.45 g N L⁻¹)* | ≥ 0.0500 g N (50.0 mg N) |
| Ammonium sulfate ((NH₄)₂SO₄) | Pure inorganic salt | 2.12 g N L⁻¹ | 0.106 g N (106 mg N) |
| Ammonium chloride (NH₄Cl) | Pure inorganic salt | 2.62 g N L⁻¹ | - 1. N (131 mg N) |

^*all nitrogen sources were supplied from Sigma-Aldrich^
